# Endicheck: Dynamic Analysis for Detecting Endianness Bugs

**DOI:** 10.1007/978-3-030-45237-7_15

**Published:** 2020-03-13

**Authors:** Roman Kápl, Pavel Parízek

**Affiliations:** 8 Johannes Kepler University, Linz, Austria; 9 University of Birmingham, Birmingham, UK; Department of Distributed and Dependable Systems, Faculty of Mathematics and Physics, Charles University, Prague, Czech Republic

## Abstract

Computers store numbers in two mutually incompatible ways: little-endian or big-endian. They differ in the order of bytes within representation of numbers. This ordering is called endianness. When two computer systems, programs or devices communicate, they must agree on which endianness to use, in order to avoid misinterpretation of numeric data values.

We present Endicheck, a dynamic analysis tool for detecting endianness bugs, which is based on the popular Valgrind framework. It helps developers to find those code locations in their program where they forgot to swap bytes properly. Endicheck requires less source code annotations than existing tools, such as Sparse used by Linux kernel developers, and it can also detect potential bugs that would only manifest if the given program was run on computer with an opposite endianness. Our approach has been evaluated and validated on the Radeon SI Linux OpenGL driver, which is known to contain endianness-related bugs, and on several open-source programs. Results of experiments show that Endicheck can successfully identify many endianness-related bugs and provide useful diagnostic messages together with the source code locations of respective bugs.

### 1 Introduction

Modern computers represent and store numbers in two mutually incompatible ways: little-endian (with the least-significant byte first) or big endian (the most-significant byte first). The byte order is also referred to as *endianness*.

Processor architectures typically define a *native endianness*, in which the processor stores all data. When two computer systems or programs exchange data (e.g., via a network), they must first agree on which endianness to use, in order to avoid misinterpretation of numeric data values. Also devices connected to computers may have control interfaces with endianness different from the host's native endianness.

Therefore, programs communicating with other computers and devices need to swap the bytes inside all numerical values to the correct endianness. We use the term *target endianness* to identify the endianness a program should use for data exchanged with a particular external entity. Note that in some cases it is not necessary to know whether the target endianness is actually little-endian or big-endian. When the knowledge is important within the given context, we use the term *concrete endianness*.

If the developer forgets to transform data into the correct target endianness, the bug can often go unnoticed for a long time because software is nowadays usually developed and tested on the little-endian x86 or ARM processor architecture. For example, if two identical programs running on a little-endian architecture communicate over the network using a big-endian protocol, a missing byte-order transformation in the same place in code will not be observed. Our work on this project was, in the first place, motivated by the following concrete manifestation of the general issue described in the previous sentence. The Linux OpenGL driver for Radeon SI graphics cards (the Mesa 17.4 version) does not work on big-endian computers due to an endianness-related bug[1], as the first author discovered when he was working on an industrial project that involved PowerPC computers in which Radeon graphic cards should be deployed.

We are aware of few approaches to detection of endianness bugs, which are based on static analysis and manually written source code annotations. An example is Sparse [11], a static analysis tool used by Linux kernel developers to identify code locations where byte-swaps are missing. The analysis performed by Sparse works basically in the same way as type checking for C programs, and relies on the usage of specialized *bitwise* data types, such as __le16 and __be32, for all variables with non-native endianness. Integers with different concrete endianness are considered by Sparse as having mutually incompatible types, and the specialized types are also not compatible with regular C integer types. In addition, macros like __le32_to_cpu are provided to enable safe conversion between values of the bitwise integer types and integer values of regular types. Such macros are specially annotated so that the analysis can recognize them, and developers are expected to use only those macros.

The biggest advantage of bitwise types is that a developer cannot assign a regular native endianness integer value to a variable of a bitwise type, or vice versa. Their nature also prevents the developer from using them in arithmetic operations, which do not work correctly on values with non-native byte order. On the other hand, a significant limitation of Sparse is that developers have to properly define the bitwise types for all data where endianness matters, and in particular to enable identification of data with concrete endianness — Sparse would produce imprecise results otherwise. Substantial manual effort is therefore required to create all the bitwise types and annotations.

Our goals in this whole project were to explore an approach based on dynamic analysis, and to reduce the amount of necessary annotations in the source code of a subject program. We present Endicheck, a dynamic analysis tool for detecting endianness bugs that is implemented as a plugin for the Valgrind framework [6]. The main purpose of the dynamic analysis performed by Endicheck is to track endianness of all data values in the running subject program and report when any data leaving the program has the wrong endianness. The primary target domain consists of programs written in C or C++, and in which developers need to explicitly deal with endianness of data values.

While the method for endianness tracking that we present is to a large degree inspired by dynamic *taint* analyses (see, e.g., [8]), our initial experiments showed that usage of existing taint analysis techniques and tools does not give good results especially with respect to precision. For example, an important limitation of the basic taint analysis, when used for endianness checking, is that it would report false positives on data that needs no byte-swapping, such as single byte-sized values. Therefore, we had to modify and extend the existing taint analysis algorithms for the purpose of endianness checking. During our work on Endicheck, we also had to solve many associated tech-

[1] https://bugs.freedesktop.org/show_bug.cgi?id=99859

nical challenges, especially regarding storage and propagation of metadata that contain the endianness information — this includes, for example, precise tracking of single-byte values.

Endicheck is meant to be used only during the development and testing phases of the software lifecycle, mainly because it incurs a substantial runtime overhead that is not adequate for production deployment. Before our Endicheck tool can be used, the subject program needs to be modified, but only to inform the analysis engine where the byte-order is being swapped and where data values are leaving the program. In C and C++ programs, byte-order swapping is typically done by macros provided in the system C library, such as htons/htonl or those defined in the endian.h header file. Thus only these macros need to be annotated. During the development of Endicheck, we redefined each of those macros such that the custom variant calls the original macro and defines necessary annotations — for examples, see Figure 1 in Section 4 and the customized header file inet.h[2]. Similarly, data also tend to leave the program only through few procedures. For some programs, the appropriate place to check for correct endianness is the send/write family of system calls.

Endicheck is released under the GPL license. Its source code is available at `https://github.com/rkapl/endicheck`.

The rest of the paper is structured as follows. Section 2 begins with a more thorough overview of the dynamic analysis used by Endicheck, and then it provides details about the way endianness information for data values are stored and propagated — this represents our main technical contribution, together with evaluation of Endicheck on the Radeon SI driver and several other real programs that is described in Section 5. Besides that, we also provide some details about the implementation of Endicheck (Section 3) together with a short user guide (Section 4).

### 2 Dynamic Analysis for Checking Endianness

We have already mentioned that the dynamic analysis used by Endicheck to detect endianness bugs is a special variant of taint analysis, since it uses and adapts some related concepts. In the rest of this paper, we use the term *endianness analysis*.

#### 2.1 Algorithm Overview

Here we present a high-level overview of the key aspects of the endianness analysis. Like common taint and data-flow analysis techniques (see, e.g., [4] and [8]), our dynamic endianness analysis tracks flow of data through program execution, together with some metadata attached to specific data values. The analysis needs to attach metadata to all memory locations for which endianness matters, and maintain them properly. Metadata associated with a sequence of bytes (memory locations) that makes a numeric data value then capture its endianness. Similarly to many dynamic analyses, the metadata are stored using a mechanism called *shadow memory* [7] [9]. We give more details about the shadow memory in Section 2.2.

[2] https://github.com/rkapl/endicheck/blob/master/endicheck/ec-overlay/arpa/inet.h

Although we mostly focus on checking that the program being analyzed does not transmit data of incorrect endianness to other parties, there is also the opposite problem: ensuring that the program does not use data of other than native endianness. For this reason, our endianness analysis could be also used to check whether all operands of an arithmetic instruction have the correct native endianness — this is important because arithmetic operations are unlikely to produce correct results otherwise. Note, however, that checking of native endianness for operands has not yet been implemented in the Endicheck tool.

The basic principle behind the dynamic endianness analysis is to watch instructions as they are being executed and check endianness at specific code locations, such as the calls of I/O functions. We use the term *I/O functions* to identify all system calls and other functions that encapsulate data exchange between a program and external entities (e.g., writing or reading data to/from a hard disk, or network communication) in a specific endianness. When the program execution reaches the call of an I/O function, Endicheck checks whether all its arguments have the proper endianness. Note that the user of Endicheck specifies the set of I/O functions by annotations (listed in Section 4).

In order to properly maintain the endianness information stored in the shadow memory, our analysis needs to track almost every instruction being executed during the run of a subject program. The analysis receives notifications about relevant events from the Valgrind dynamic analysis engine. All the necessary code for tracking individual instructions (processing the corresponding events), updating endianness metadata (inside the shadow memory), and checking endianness at the call sites of I/O functions, is added to the subject program through dynamic binary instrumentation. Further technical details about the integration of Endicheck into Valgrind are provided later in Section 3.

Two distinguishing aspects of the endianness analysis — the format of metadata stored in the shadow memory and the way metadata are propagated during the analysis of program execution — are described in the following subsections.

#### 2.2 Shadow Memory

A very important requirement on the organization and structure of shadow memory was full transparency for any C/C++ or machine code program. The original layout of heap and stack has to be preserved during the analysis run, since Endicheck (and Valgrind in general) targets C and C++ programs that typically rely on the precise layout of data structures in memory. Consequently, Endicheck cannot allocate the space for shadow memory (metadata) within the data structures of the analyzed program.

When designing the endianness analysis, we decided to use the mechanism supported by Valgrind [7], which allows client analyses to store a tag for each byte in the virtual address space of the analyzed program without changing its memory layout. This mechanism keeps a translation table (similar to page tables used by operating systems) that maps memory pages to *shadow pages* where the metadata are stored.

The naive approach would be to follow the same principles as taint analyses, i.e. reuse the idea of taint bits, and mark each byte of memory as being either of native endianness or target endianness. However, our endianness analysis actually uses a richer format of metadata and individual tags, which improves the analysis precision.

**Rich Metadata Format.** In this format of metadata, each byte of memory and each processor register is annotated with one of the following tags that represent available knowledge about the endianness of stored data values.

- **native**: The default endianness produced, for example, by arithmetic operations.
- **target**: Used for data produced by annotated byte-swapping function.
- **byte-sized**: Marks the first byte of a multi-byte value (e.g., an integer or float).
- **unknown**: Endianness of uninitialized data (e.g., newly allocated memory blocks).

In addition to these four tags, each byte of memory can also be annotated with the **empty** flag, indicating that the byte's value is zero. Now we give more details about the meaning of these tags, and discuss some of the associated challenges.

**Single-byte values.** Our approach to precise handling of single-byte values is motivated by the way arithmetic operations are processed. Determining the correct size of the result of an arithmetic operation (in terms of the number of actually used bytes) is difficult in practice, because compilers often choose to use instructions that operate on wider types than actually specified by the developer in program source code. This means the analysis cannot, in some cases, precisely determine whether the result of an arithmetic operation has only a single byte. Our solution is to always mark the least-significant byte of the result with the tag byte-sized. Such an approach guarantees that if only the least-significant byte of an integer value is actually used, it does not trigger any endianness errors when checked, because the respective memory location is not tagged as native. On the other hand, if the whole integer value is really used (or at least more than just the least-significant byte), there is one byte marked with the tag byte-sized and the rest of the bytes are marked as native, thus causing an endianness error when checked.

**Empty byte flag.** Usage of the empty flag helps to improve performance of the endianness analysis when processing byte-shuffling instructions, because all operations with empty flags are simpler than operations with the actual values. However, this flag can be soundly used only when the operands are byte-wise disjoint, i.e. when each byte is zero (empty) in at least one of the operands. Arithmetic operations are handled in a simplified way — they never mark bytes as empty in the result. Consequently, while the empty tag implies that the given byte is zero, the reverse implication does not hold.

**Unknown tag.** We introduced the tag unknown in order to better handle data values, for which the analysis cannot say whether they are already byte-swapped. Endicheck uses this tag especially for uninitialized data. Values marked with the tag unknown are not reported as erroneous by default, but this behavior is configurable. We discuss other related problems, concerning especially precision, below in Section 2.4.

#### 2.3 Propagation of Metadata

An important aspect of the endianness analysis is that data values produced by the subject program are marked as having the native endianness by default. This behavior matches the prevailing case, because data produced by most instructions (e.g., by arithmetic operations) and constant values can be assumed to have native endianness.

In general, metadata are propagated upon execution of an instruction according to the following policy:

- Arithmetic operations always produce native-endianness result values.
- Data manipulation operations (e.g., load and store) propagate tags from their operands to results without any changes.

Endicheck correctly passes metadata also through routines such as memcpy and certain byte-shuffling operations (e.g., shift <<= and >>=). Complete details for all categories of instructions and routines are provided in the master thesis of the first author [3].

The only way to create data with the target tag is via explicit annotation from the user. Specifically, the user needs to add annotations to *byte-swapping* functions in order to set the target tag on return values.

#### 2.4 Discussion: Analysis Design and Precision

The basic scenario that is obviously supported by our analysis is the detection of endianness bugs when the target and native endianness are different. However, the design of our analysis ensures that it can be useful even in cases when the native endianness is the same as the target endianness. Although byte-swapping functions then become identities, the endianness analysis can still find data that would not be byte-swapped if the endianities were different — it can do this by setting the respective tags when data pass through the byte-swapping functions. In addition, the endianness analysis can be also used to detect the opposite direction of errors — programs using non-native endianness data values (e.g., received as input) without byte-swapping them first.

Endicheck does not handle constants and immediate values in instructions very well, since the analysis cannot automatically recognize their endianness and therefore cannot determine whether the data need byte-swapping or not. Constants stored in the data section of a binary executable represent the main practical problem to the analysis, because the data section does not have any structure — it is just a stream of bytes. Our solution is to mark data sections initially with the tag unknown. If this is not sufficient, a user must annotate the constants in the program source code to indicate whether they already have the correct endianness.

A possible source of false bug reports are unused bytes within a block of memory that has undefined content, unless the memory was cleared with 0s right after its allocation. This may occur, for example, when some fields inside C structures have specific alignment requirements. Some space between individual fields inside the structure layout is then unused, and marked either with the tag unknown or with the tag left over from the previous content of the memory block.

### 3 Implementation

We distribute the Endicheck tool in the form of an open source software package that was initially created as a fork of the Valgrind source code repository. Although tools and plugins for Valgrind can be maintained as separate projects, forking allowed us to make changes to the Valgrind core and use its build/test infrastructure. Within the whole source tree of Endicheck, which includes the forked Valgrind codebase, the code specific to Endicheck is located in the endicheck directory. It consists of these modules:

- ec_main: tool initialization, command-line handling and routines for translation to/from intermediate representation;
- ec_errors: error reporting, formatting and deduplication;
- ec_shadow: management of the shadow memory, storing of the endianness metadata, protection status and origin tracking information (see below);
- ec_util: utility functions for general use and for manipulation with the metadata;
- endicheck.h: public API with annotations to be used in programs by developers.

In the rest of this section, we briefly describe how Endicheck uses the Valgrind infrastructure and a few other important features. Additional technical details about the implementation are provided in the master thesis of the first author [3].

**Usage of Valgrind infrastructure.** Endicheck depends on the Valgrind core (i) for dynamic just-in-time instrumentation [6] of a target binary program and (ii) for the actual dynamic analysis of program execution. The subject binary program is instrumented with code that carries out all the tasks required by our endianness analysis — especially recording of important events and tracking information about the endianness of data values. When implementing the Endicheck plugin, we only had to provide code doing the instrumentation itself and define what code has to be injected at certain locations in the subject program. Note also that for the analysis to work correctly and provide accurate results, Valgrind instruments all components of the subject program that may possibly handle byte-swapped data, including application code, the system C library and other libraries. During the analysis run, Valgrind notifies the Endicheck plugin about execution of relevant instructions and Endicheck updates the information about endianness of affected data values accordingly. Besides instrumentation and the actual dynamic analysis, other features and mechanisms of the Valgrind framework used by Endicheck include: utility functions, origin tracking, and developer-friendly error reporting.

Origin tracking [1] is a mechanism that can help users in debugging the endianness issues. An error report contains two stack traces: one identifies the source code location of the call to the I/O function where the wrong endianness of some data value was detected, and the second trace, provided by origin tracking, identifies the source code location where the value has originated. In Endicheck, the origin information (identifier of the stack trace and execution context) is stored alongside the other metadata in the shadow memory for all values. We decided to use this approach because almost all values need origin tracking, since they can be sources of errors — in contrast to Memcheck, where only the uninitialized values can be sources of errors.

During our experiments with the Radeon SI OpenGL driver (described in Section 5.1), we have noticed that the driver maps the device memory into the user-space process. In that case, there is no single obvious point where to check the endianness of data that leave the program through the mapped memory. To solve this problem and support memory-mapped I/O, we extended our analysis to automatically check endianness at all writes to regions of the mapped device memory. We implemented this feature in such a way that each byte of a device memory region is tagged with a special flag protected — then, Endicheck can find very quickly whether some region of memory is mapped to a device or not. Note that the flag is associated with a memory location, while the endianness tags (described in Section 2.2) are associated with *data values*. Therefore, the special flag is not copied, e.g. when execution of memcpy is analyzed; it can be only set explicitly by the user.

### 4 User Guide

The recommended way to install Endicheck is building from the source code. Instructions are provided in the README file at the project web site. When Endicheck has been installed, a user can run it by executing the following command:

```
valgrind --tool=endicheck [OPTIONS...] PROGRAM ARGS...
```

Origin tracking is enabled by the option –track-origins=yes.

**Annotations** In order to analyze a given program, some annotations typically must be added into the program source code. A user of Endicheck has to mark the byte-swapping functions and the I/O functions (through which data values are leaving the program), because these functions cannot be reliably detected in an automated way.

The specific annotations are defined in the C header file endicheck.h. Here follows the list of supported annotations, together with explanation of their meaning:

- EC_MARK_ENDIANITY(start, size, endianness)
  This annotation marks a region of memory from start to start+size-1 as having the given endianness. It should be used in byte-swapping functions. Target endianness is represented by the symbol EC_TARGET.
- EC_CHECK_ENDIANITY(start, size, msg)
  This annotation enforces a check that a memory region from start to start+size-1 contains only data with *any* or *target* endianness. It should be used in I/O functions. *Unknown* endianness is allowed by passing the –allow-unknown option.
- EC_PROTECT_REGION(start, size)
  Marks the given region of memory as protected. This should be used for mapped regions of device memory.
- EC_UNPROTECT_REGION(start, size)
  Marks the given memory region as unprotected.
- EC_DUMP_MEM(start, size)
  Dumps endianness of a memory region. This is useful for debugging.

Figure 1 shows an example program that demonstrates usage of the most important annotations (EC_MARK and EC_CHECK). If the call to htobe32 inside main is removed, Endicheck will report an endianness bug. This example also demonstrates possible ways to easily annotate standard functions, like htobe32 and write.

```
#include <valgrind/endicheck.h>

uint32_t htobe32(uint32_t x) {
#if __BYTE_ORDER__ == __ORDER_LITTLE_ENDIAN__
    x = bswap_32(x);
#endif
    EC_MARK_ENDIANITY(&x, sizeof(x), EC_TARGET);
    return x;
}

int ec_write(int file, const void *buf, size_t count) {
    EC_CHECK_ENDIANITY(buf, count, NULL);
    return write(file, buf, count);
}
#define write ec_write int main() {
   uint32_t x = 0xDEADBEEF;
   x = htobe32(x);
   write(0, &x, sizeof(x));
   return 0;
}
```

**Fig. 1.** Small example program with Endicheck annotations.

## 5 Evaluation

We evaluated the Endicheck tool — namely its ability to find endianness bugs, precision and overhead — by the means of a case study on the Radeon SI driver, several open-source programs and a standardized performance benchmark. For the Radeon SI driver and each of the open-source programs, we provide a link to its source code repository (and identification of the specific version that we used for our evaluation) within the artifact that is referenced from the project web site.

### 5.1 Case Study

Our case study is Radeon SI, the Linux OpenGL driver for Radeon graphics cards, starting with the SI (Southern Islands) line of cards and continuing to the current models.

Since these Radeon cards are little-endian, the driver must byte-swap all data when running on a big-endian architecture such as PowerPC. However, the Radeon SI driver (in the Mesa 17.4 version) does not perform the necessary byte-swapping operations, and therefore simply does not work in the case of PowerPC — it crashes either the GPU or OpenGL programs using the driver. In particular, endianness bugs in this version of the Radeon SI driver cause the Glxgears demo on PowerPC to crash. We give more details about the bugs we have found in Section 5.2.

An important feature of the whole Linux OpenGL stack is that all layers, including the user-space program, communicate not only using calls of library functions and system calls, but they also extensively use mapping of the device memory directly into the user process. Given such an environment, Endicheck has to correctly handle (1) the flow of data through the whole OpenGL stack by instrumenting all the libraries used, and (2) communication through the shared memory that is used by the driver. This is why the support for mapped memory in Endicheck, through marking of device memory with a special flag, as described above in Section 3, is essential.

### 5.2 Search for Bugs

For the purpose of evaluating Endicheck's ability to find endianness bugs, we picked a diverse set of open-source programs (in addition to the Radeon SI driver), including the following: BusyBox, OpenTTD, X.Org and ImageMagick. All programs are listed in Table 1. The only criterion was to select programs written in C that communicate over the network or store data in binary files, since only such programs may possibly contain endianness bugs. We also document our experience with fixing the endianness bugs in the Radeon SI driver and other programs.

One of the stated goals for Endicheck was to reduce the number of annotations that a user must add into the program source code in order to enable search for endianness bugs. Therefore, below we report the relevant measurements and discuss whether (and to what degree) this goal has been achieved.

In the rest of this section, first we discuss application of Endicheck on the Radeon SI driver (our case study) and then we present results for other programs.

**Radeon SI case study.** Within our case study, we have used the Glxgears demo program as a test harness for the Radeon SI driver. Initially we have run Glxgears on the x86 architecture, and after fixing all the issues found and reported by Endicheck, we moved the same graphics card to a PowerPC host computer and continued testing there.

In the case of the Radeon SI driver, all byte-swapping functions are located in a single file of one library (Gallium) on the OpenGL stack. Therefore, to enable search for endianness bugs in Radeon SI, we had to make just two changes: (1) annotate the function radeon_drm_cs_add_buffer as I/O function and (2) annotate the byte-swapping functions in Gallium. Overall, we had to add or change about 40 lines of source code, including annotations, in a single place. All our changes are published in the repository `https://rkapl.cz/repos/git/roman/mesa`. It contains the source code of Mesa augmented with our annotations and fixes for the endianness-related bugs in Radeon SI described below. For fixes of bugs found by Endicheck, we included the original Endicheck report in the commit message, under the ECNOTE header.

Figure 2 contains an example bug report produced by Endicheck with enabled origin tracking on Glxgears. The error report itself has three main parts (in this order): the problem description, origin stack trace (captured when the offending value is created) and point-of-check stack trace (recorded when some annotated I/O function is encountered). We show only fragments of stack traces for illustration (and to save space).

The problem description identifies the currently active thread, the nature of the error and the memory region containing the erroneous value. The memory region is identified by its address and an optional name provided by the program ("radeon_add_buffer" in

```
Thread 9 gallium_drv:0:
Memory does not contain data of Target endianness
Problem was found in block 0x41BF000 (named radeon_add_buffer)
at offset 0, size 8:
   0x41BF000: N N N N N N N N
The value was probably created at this point:
   at 0x8B787F7: si_init_msaa_functions (si_state_msaa.c:94)
   by 0x8B4F979: si_create_context (si_pipe.c:279)
      ...
   by 0x4C46661: glXCreateContext (glxcmds.c:427)
   by 0x10B67A: make_window.constprop.1 (glxgears.c:559)
   by 0x109A86: main (glxgears.c:777)
The endianness check was requested here:
   at 0x8B85C45: radeon_drm_cs_add_buffer (radeon_drm_cs.c:375)
   by 0x8B4A58B: si_set_constant_buffer (r600_cs.h:74)
   by 0x8B708D0: si_set_framebuffer_state (si_state.c:2934)
      ...
   by 0x55357FB: start_thread (pthread_create.c:465)
   by 0x5861B0E: clone (clone.S:95)
```

**Fig. 2.** Error report from Endicheck run on the Glxgears demo program this case). Metadata are printed just for the part of the memory region that contains data with the wrong endianness, using this convention: N = native, U = undefined.

This particular error report (Figure 2) indicates that an array of floating-point values describing the *multisampling pattern* is not byte-swapped. Note that IEEE 754 floating point values also obey the endianness of the host platform, at least on the architectures x86, x64 and ARM. To repair the corresponding bug, we had to insert calls of byte-swapping functions at the code location where the floating-point array is produced.

During our experiments with Radeon SI and Glxgears, four endianness bugs in total were detected by Endicheck on the x86 architecture before testing on PowerPC. After we fixed the bugs, the Glxgears demo did successfully run. This shows that Endicheck detected all bugs it was supposed to and provided reports useful enough so that the bugs could be fixed. Here we also need to emphasize that the Glxgears demo, naturally, does not exercise all code in the Radeon SI driver, and fixing the whole driver would require lot of additional work.

**Other programs.** As we said at the beginning of this section, we evaluated Endicheck's ability to find endianness bugs and precision on a set of realistic programs. Our primary goal in this part of the evaluation was to assess the following aspects:

- the extent of annotations that is required for Endicheck to work properly,
- whether Endicheck is able to detect a bug in a given kind of programs, and
- how many false warnings are reported.

Before trying to answer these questions, we wanted to be sure that the subject programs contain endianness bugs. However, some of the programs that we considered

(OpenTTD, OpenArena and ImageMagick) are written in such a way that realistic endianness bugs cannot be injected into their codebase. ImageMagick uses a C++ abstraction layer for binary streams, which also handles endianness. OpenArena uses bit-oriented encoding for most parts of the network communication. OpenTTD uses an abstraction layer too, but the developer can still make an endianness-related mistake in certain cases, such as storing an array of uint16_t values as an array of uint8_t values. We manually injected synthetic endianness bugs into the code of all the programs where this was possible. In this process, we also annotated the byte-swapping functions (like htonl). The bugs were created by removing one usage of byte-swapping functions.

The results of experiments are summarized in Table 1. For each program, the table provides the following information: whether it was possible to analyze the program at all, whether some endianness bugs were found, overhead related to false warnings, and how many lines of source code were added or changed in relation to Endicheck annotations. Data for the Radeon SI driver are also included in the table for completeness.

| Program | Analyzable | Injected bug | False positives | Actual bugs | Annotations |
|---|---|---|---|---|---|
| Radeon SI driver | ✓Yes | ✓Found | ∅Manageable (2) | ✓Found | cca 40 lines |
| BusyBox | ✓Yes | ✓Found | ✓No | None found | 20 lines |
| OpenTTD | ✓Partially | ✓Found | ∅Manageable (2) | None found | 59 lines |
| Ntpd | ✓Yes | ✓Found | ✓No | None found | 1 line |
| X.Org | ✓Yes | ✓Found | ✓No | ✓Found | 30 lines |
| OpenArena | ∅No | | | | |
| ImageMagick | ∅No | | | | |

**Table 1.** Search for bugs: precision and necessary annotations

Data in Table 1 show that Endicheck could find the introduced bug in all the cases. Furthermore, Endicheck found two genuine endianness-related bugs in X.Org. The bugs were confirmed by the developers of X.Org and fixed in upstream[3].

Endicheck also reports some false warnings, but their numbers are not overwhelming. Four cases in total occured for the Radeon SI driver and OpenTTD (two in each). This is a manageable amount, which can be even suppressed using further annotations.

#### 5.3 Performance

In this section, we report on the performance of Endicheck in terms of execution time overhead it introduces. We compare the performance data for programs instrumented with Endicheck, programs instrumented by the Memcheck plugin for Valgrind and programs without any instrumentation. For the purpose of experiments, we used the standardized benchmark SPEC CPU2000. Even though SPEC CPU2000 is a general benchmark, not tailored for endianness analysis, results of experiments with this benchmark

[3] https://gitlab.freedesktop.org/search?group_id=&project_id=371&repository_ref=master&scope=commits&search=Roman+Kapl indicate the performance of Endicheck when doing a real analysis, because the control-flow paths exercised within Endicheck and the Valgrind core during an experiment do not depend on the specific metadata (tag values).

We run all experiments on a T550 ThinkPad notebook with 12 GiB of RAM and an i5-5200 processor clocked at 2.20 GHz, under Arch Linux from Q2 2018. The SPEC2000 test harness was used for all the runs, with iteration count set to 3. We compiled both Memcheck and Endicheck by GCC v7.3.0 with default options. Note that we had to omit the benchmark program "gap", because it produced invalid results when compiled with this version of GCC.

In the description of specific experiments, tables with results and their discussion, we use the following abbreviations:

- **EC**: Endicheck (valgrind –tool=endicheck)
- **MC**: Memcheck (valgrind –tool=memcheck)
- **-OT**: with precise origin tracking enabled (–track-origins=yes)
- **-IT**: with origin tracking enabled, but not fully precise (–precise-origins=no)
- **-P**: with memory protection enabled (–protection=yes)

**Execution time.** We divided our experiments designed for measuring the execution time into two groups. Our motivation was to ensure that all experiments, including the EC-OT configuration that incurs a large overhead, finish within a reasonable time limit. In the first group, we run the full range of configurations on the "test" data set provided by SPEC CPU2000, which is small compared to the full "reference" set, and used MC as the baseline for comparisons. Table 2 shows results for experiments in this group. All execution time data provided in this table are relative to MC, with the exception of data for the native configuration. The second group of experiments uses the full "reference" data set from SPEC CPU2000. Results for this group are provided in Table 3. In this case, we used the data for native (uninstrumented) programs as the baseline.

| Program | Native (s) | MC (s) | MC-OT | EC | EC-P | EC-OT | EC-IT |
|---|---|---|---|---|---|---|---|
| bzip2 | 1.38 | 19.40 | 2.27x | 2.07x | 2.23x | 33.87x | 12.58x |
| crafty | 0.70 | 18.70 | 2.21x | 1.74x | 1.78x | 30.59x | 11.07x |
| eon | 0.09 | 6.60 | 1.73x | 1.29x | 1.34x | 12.89x | 4.23x |
| gcc | 0.31 | 12.70 | 1.96x | 1.92x | 1.98x | 24.17x | 9.53x |
| gzip | 0.47 | 6.29 | 2.11x | 1.86x | 1.97x | 41.97x | 14.96x |
| mcf | 0.05 | 0.85 | 2.38x | 1.27x | 1.32x | 11.88x | 7.08x |
| parser | 0.66 | 10.50 | 2.19x | 2.13x | 2.28x | 41.24x | 16.29x |
| perlbmk | 4.31 | 5.52 | 1.10x | 0.95x | 0.95x | 1.17x | 1.05x |
| twolf | 0.05 | 1.64 | 1.88x | 1.16x | 1.20x | 14.09x | 5.51x |
| vortex | 1.06 | 56.90 | 2.23x | 1.95x | 2.04x | 28.38x | 9.86x |
| vpr | 0.49 | 8.02 | 2.00x | 1.70x | 1.75x | 22.94x | 8.30x |
| G.mean | 0.41 | 7.86 | 1.97x | 1.59x | 1.65x | 18.17x | 7.56x |

**Table 2.** Execution times for the SPEC CPU2000 test data set, relative to Memcheck.

| Program | Native (s) | MC | EC | EC-P |
|---|---|---|---|---|
| bzip2 | 66.3 | 11.63x | 23.47x | 24.45x |
| crafty | 29.5 | 26.78x | 48.10x | 48.54x |
| eon | 24.1 | 52.12x | 93.36x | 97.34x |
| gcc | 27.8 | 27.73x | 116.62x | 122.48x |
| gzip | 79.9 | 8.92x | 15.93x | 16.80x |
| mcf | 67.10 | 2.71x | 6.90x | 6.94x |
| parser | 89.9 | 10.78x | 23.04x | 23.86x |
| perlbmk | 45.9 | 38.45x | 93.62x | 96.27x |
| twolf | 93 | 12.43x | 19.77x | 19.52x |
| vortex | 43.8 | 44.36x | 91.03x | 92.85x |
| vpr | 54.7 | 10.49x | 20.29x | 20.68x |
| G.mean | 51.29 | 16.59x | 35.31x | 36.25x |

**Table 3.** Execution times for the SPEC CPU2000 reference data set, relative to native runs.

Data in Table 3 indicate that the average slowdown of Memcheck is by the factor of 16.59. Endicheck, in comparison, slows down the analyzed program by the factor of 35.31. This means Endicheck has roughly two times higher overhead than Memcheck with default options. According to data in Table 2, the same relative slowdown of Endicheck with respect to Memcheck is 1.65x. This difference between the results for the reference and test data sets is caused by the different ratio of the time spent instrumenting the code versus time spent running the instrumented code.

However, data in both tables also show that the performance of Endicheck with origin tracking is lacking compared to Memcheck with the same option. It was still usable for our Radeon SI OpenGL tests, but measurements indicate that there is a space for optimization. Nevertheless, certain relative slowdown between the configurations EC-OT and MC-OT probably cannot be avoided, because Endicheck must track origin information for much more data than Memcheck. Based on our experiments, we observed that creating the origin information is the most expensive operation involved. When the origin tags are created for each superblock, instead of every instruction, the execution times drop roughly by a factor of two (see the columns EC-OT and EC-IT).

#### 5.4 Discussion

Based on the case study and results of experiments presented in the previous sections, we make the following general conclusions:

- Endicheck can find true endianness bugs in large real programs, assuming that the user correctly annotates all the byte-swapping functions and I/O functions.
- Using fairly complex metadata is feasible in terms of performance and encoding.
- Performance of Endicheck is practical even on large programs, despite the overhead and given that its current version is not yet optimized as well as Memcheck.
- Although Endicheck, due to precise dynamic analysis, requires less annotations to be specified manually than static analysis-based tools (e.g., Sparse), still it puts certain burden on the user.

Regarding the annotation burden, we already mentioned that the user has to carefully mark in particular all the I/O functions and byte-swapping functions, so that Endicheck can correctly update endianness tags associated with memory locations during the run of the analysis. While it would be possible to recognize byte-swapping functions automatically, e.g. by static code analysis, then the endianness analysis would have to be run on a machine with the native endianness different from the target endianness, so that actual byte-swaps will be present.

Another limitation of Endicheck from the practical perspective is handling of complex data transformations, a problem shared with taint analysis. The metadata cannot be correctly preserved through transformations such as encryption/decryption and compression/decompression. However, in many cases, the problem could be avoided by requiring an endianness check to be performed just before the respective transformation.

### 6 Related Work

As far as we know, the Sparse tool [11] used by Linux kernel developers, which we already mentioned, is the only one publicly available specialized tool tackling the problem of finding endianness bugs. The main advantage of Endicheck over Sparse is better precision in some cases, i.e. fewer false bug reports, since dynamic analysis, which observes actual program execution and runtime data values, is typically more precise than static analysis. Endicheck also does not require so many annotations of functions and variables as Sparse — when using Endicheck, typically just few places in the program source code need to be annotated manually. More specifically, Sparse expects that an input program code involves (i) the specialized bitwise data types (e.g., `__le32`) for all variables where endianness matters and (ii) the macros for conversion between regular types and bitwise types (e.g., `__le32_to_cpu`). With Endicheck, developers only have to annotate the byte-swapping functions used by the program (e.g., `htons` and `htonl` from the C library). On the other hand, Sparse has better coverage of program code, as it is based on static analysis.

The Valgrind dynamic analysis framework [6] comes bundled with a set of bug detection tools. Very popular is the Memcheck tool [5] for detecting memory access errors and leaks, which also served as an inspiration for the design and implementation of Endicheck. We mention the tool here, because it actually performs a variant of dynamic taint analysis — it marks each bit of the program memory as valid or invalid (tainted).

Closely related is also the runtime type checker Hobbes [2] for binary executables, which can detect some kinds of type mismatch bugs common in C programs. In order to reduce the number of false bug reports and to delimit integer values, Hobbes uses the mechanism of continuation markers — the first byte of each value has the marker unset, and the remaining bytes are set to indicate that they represent a continuation of an existing value. The analysis technique used by Hobbes could be modified to track endianness of integer values instead of distinguishing between pointers and integers, since one can model integers of different endianness as values that have different types (also like in the case of Sparse).

Another approach with functionality similar to Endicheck has been implemented within the LLVM/Clang plugin called DataFlowSanitizer [10]. It is a dynamic analysis framework that (i) enables programs to define tags for data values and check for specific tags, both through its API functions, and (ii) propagates all tags with the data.

## 7 Conclusion

We have presented a new dynamic analysis tool, Endicheck, for detecting endianness bugs in C/C++ programs. The tool is built upon the Valgrind framework. Endicheck provides a useful, and in many settings also preferable, alternative to static analysis tools like Sparse, because (1) it reports quite precise results (i.e., a low number of false warnings) due to the nature of dynamic analysis and (2) requires less annotations (and other changes) in the source code of the subject program in order to be able to detect missing byte-swap operations. The results of our experimental evaluation show that Endicheck can (1) handle large complex programs and (2) identify actual endianness bugs, and it has practical performance overhead. Endicheck could also be used in automated testing scenarios, as a useful alternative to testing programs on both little- and big-endian processor architecture. A testing environment based on Endicheck might be easier to set-up than the environment based, for example, on virtual machines.

### 7.1 Future Work

Possible extensions of Endicheck, which could improve its precision and practical usefulness even further, include:

- More complex analysis approach based on explicit tagging of each byte in an integer data value with its position.
- Reporting arithmetic instructions that use data with target endianness.
- Automatically checking system calls such as `write` for correct endianness.
- Suppression files for endianness bug reports to eliminate false positives.

Another way to detect endianness bugs more precisely is to use comparative runs (i.e, a kind of equivalence checking). The key idea is to run a program on two machines, where one has a big-endian architecture and the other has a little-endian architecture, and compare the data leaving both variants of the program. This approach has the potential to be the most accurate, because it can even detect problems in cases when data leaving the program are encrypted or compressed. On the other hand, it cannot always detect situations when the program forgets to byte-swap input data, unless the error affects one of the output values with *concrete* endianness.

**Acknowledgments.** This work was partially supported by the Czech Science Foundation project 18-17403S and partially supported by the Charles University institutional funding project SVV 260451.

## References

[1] Bond, M.D., Nethercote, N., Kent, S.W., Guyer, S.Z., McKinley, K.S.: Tracking Bad Apples: Reporting the Origin of Null and Undefined Value Errors. In: Proceedings of OOPSLA 2007. ACM (2007)

[2] Burrows, M., Freund, S.N., Wiener, J.L.: Run-Time Type Checking for Binary Programs. In: Proceedings of CC 2003. LNCS, vol. 2622. Springer (2003)

[3] Kapl, R.: Dynamic Analysis for Finding Endianity Bugs. Master thesis, Charles University, Prague, June 2018

[4] Liu, Y., Milanova, A.: Static Analysis for Inference of Explicit Information Flow. In: Proceedings of PASTE 2008. ACM (2008)

[5] Seward, J., Nethercote, N.: Using Valgrind to Detect Undefined Value Errors with Bit-Precision. In: Proceedings of USENIX 2005 Annual Technical Conference. USENIX Association (2005)

[6] Nethercote, N., Seward, J.: Valgrind: A Framework for Heavyweight Dynamic Binary Instrumentation. In: Proceedings of PLDI 2007. ACM (2007)

[7] Nethercote, N., Seward, J.: How to Shadow Every Byte of Memory Used by a Program. In: Proceedings of VEE 2007. ACM (2007)

[8] Newsome, J., Song, D.: Dynamic Taint Analysis for Automatic Detection, Analysis, and Signature Generation of Exploits on Commodity Software. In: Proceedings of NDSS 2005. The Internet Society (2005)

[9] Serebryany, K., Bruening, D., Potapenko, A., Vyukov, D.: AddressSanitizer: A Fast Address Sanity Checker. In: Proceedings of USENIX 2012 Annual Technical Conference. USENIX Association (2012)

[10] Clang 8 documentation / DataFlowSanitizer. https://clang.llvm.org/docs/DataFlowSanitizer.html (accessed in October 2019)

[11] Sparse: a semantic parser for C programs. https://lwn.net/Articles/689907/ (accessed in October 2019)

